# Neutralizing antibody landscape of the non-polio Enteroviruses and future strategy

**DOI:** 10.3389/fimmu.2024.1524356

**Published:** 2025-01-14

**Authors:** Hongye Wang, Wenbing Zhu, Ya Li, Ming Sun

**Affiliations:** ^1^ Institute of Medical Biology, Chinese Academy of Medical Sciences and Peking Union Medical College, Kunming, China; ^2^ Department of Laboratory Medicine, The Affiliated Hospital of Yunnan University, Kunming, China

**Keywords:** *Enterovirus*, neutralizing antibody, mechanism, breadth, potency

## Abstract

The non-polio Enteroviruses (NPEVs), consist of enteroviruses, coxsackieviruses, echoviruses, and rhinoviruses, are causative agents for a wide variety of diseases, ranging from common cold to encephalitis and acute flaccid paralysis (AFP). In recent years, several NPEVs have become serious public health threats, include EV-A71, which has caused epidemics of hand-foot-and-mouth disease (HMFD) in Southeast Asia, and EV-D68, which caused outbreaks of severe respiratory disease in children worldwide. Infections with these viruses are associated with neurological diseases like aseptic meningitis and AFP. Currently, apart from inactivated EV-A71 vaccines that were developed in China, no effective measures are available to prevent or treat NPEV infections. Antibody-mediated immunity is crucial for preventing and limiting viral infections, and potent neutralizing antibodies could serve as potential therapeutic agents. In this review, we describe recent progress in the NPEVs neutralization antibodies, summarizing the characteristics, breadth, and potency against NPEVs, such as EV-A71, CVA16, EV-D68, and echovirus. We focus on not only through the study of viral epitopes but also through the understanding of virus-antibody interactions. Also, we decipher the role of antibodies in the attachment of the virus to receptors, internalization, and uncoating process, providing insight into virus neutralization mechanisms. Moreover, bi-specific antibodies or multivalent antibodies with better potency are also discussed. Therefore, an in-depth understanding of structures of enterovirus and mechanisms of antibody neutralization should be useful for future strategies in guiding the design of a rational antiviral agent against NPEVs infections.

## 1 Introduction

The *Enterovirus* genus is one of the most prevalent genera in the *Picornavirus* family, of which species that infect human consists of polio virus, coxsackieviruses, numbered enteroviruses, echoviruses, and rhinoviruses. These different viruses cause a variety of diseases, including common cold, herpangina, hand, foot and mouth disease (HFMD), encephalitis, neonatal sepsis like disease, and acute flaccid paralysis (AFP). Among these viruses, poliovirus which causes AFP is the most famous enterovirus and has been intensively investigated since its identification in 1908. In recent years, several non-polio Enteroviruses (NPEVs) cause a range of epidemics and have become serious public health threats (1, 2), including enterovirus A71 (EV-A71), which has caused epidemics of hand-foot-and-mouth disease (HMFD) in Southeast Asia (3, 4), and EV-D68, which caused outbreaks of severe respiratory disease in children worldwide (5–8), and CVA24 variant, which lead to large outbreaks of acute hemorrhagic conjunctivitis that affects millions of people (9, 10).

To avoid inconsistencies in the nomenclature that lead to puzzling problems, the International Committee for Taxonomy of Viruses (ICTV) Picornaviridae Study Group corrected the use of taxon names for *Enterovirus* and *Rhinovirus* viruses, the *Enterovirus* were thus divided into *Enterovirus A* to *Enterovirus D* (EV-A, B, C, and D), and *Rhinovirus* divided into *Rhinovirus A* to *Rhinovirus C* (RV-A, B, and C) (11). EV-As include some CV-A types and some numbered EVs, and are the main pathogenic pathogens that cause hand foot mouth disease (HFMD), including enterovirus A71 (EV-A71), coxsackievirus A16 (CVA16), CVA6, and CVA10 (12–16). EV-Bs include CVB types, CVA9 and echoviruses. EV-Cs include poliovirus, some numbered EVs and several CVA types. EV-Ds include some numbered EVs, among which EV-D68 is the most known that cause outbreaks. In addition, enteroviruses have high rates of mutation and genomic recombination, and the chances of co-infection also increased the possibility of generating new pathogenic strains (17, 18).

Currently, apart from inactivated EV-A71 vaccines that were developed in China (19–21), no effective measures are available to prevent or treat NPEV infections. The EV-A71 vaccines induced weak cross-neutralization response against CVA16, and hence are not applicable for preventing CVA16 and other enterovirus-associated HFMD (22), development of multivalent enterovirus vaccines that use different strategies such as DNA/RNA vaccine technology could benefit for faster and broad protection (23–25). Antibody-mediated immunity is crucial for preventing and limiting viral infections, and potent neutralizing antibodies could serve as potential therapeutic agents for enterovirus infections. To date, there are only a few anti-EV monoclonal neutralizing antibodies identified, and the cross-reactivity of most antibodies remains to be enhanced. The majority of the substantial neutralizing sites are in the capsid of NPEVs, mainly in VP1, VP2, and VP3 of the capsid, which are exposed on the surface (26, 27). A deeper understanding of the epitope, cross-reactivity, and neutralizing mechanism of NPEVs could benefit in future design and development of vaccines and therapeutics.

In this review, we discuss identified monoclonal antibodies that target different NPEVs, viral epitopes and virus-antibody interactions were depicted. Also, we decipher the role of antibodies in the attachment of the virus to receptors, internalization, and uncoating process, providing insight into virus neutralization mechanisms. Moreover, bi-specific antibodies or multivalent antibodies with better potency are also discussed. Therefore, an in-depth understanding of structures of enterovirus and mechanisms of antibody neutralization should be useful for future strategies in guiding the design of a rational antiviral agent against NPEVs infections.

## 2 Anti-*Enterovirus A* antibodies

EV-A71 which has caused epidemics of hand-foot-and-mouth disease (HMFD) is the most studied serotype of Enterovirus A. The entry of EV-A71 into host cells is facilitated mainly through the endocytic pathway, which involves attachment to host-specific cell receptors, internalization, uncoating, and genome releasing into the cytoplasm. Virus attachment with a specific receptor, mostly are heparan sulfate (HS) (28–30), P-selectin glycoprotein ligand-1 (PSGL-1 or CD162) (31–33), and Human scavenger receptor class B member 2 (SCARB2) (34–37), the initial key step of infection, is an interaction target for developing antiviral therapeutics through antibody-receptor-virus interactions.

Antibodies against EV-A71 could neutralize the virus by inhibiting receptor binding. The monoclonal antibody MA28-7 neutralizes EV-A71 by competing with PSGL-1 and HS during initial attachment and binding to a conserved glycine at amino acid VP1-145 across the 5-fold symmetry axes (38). In suckling BALB/c mice, MAb BB1A5 (60 µg/g) conferred 100% passive protection againstEV-A71 infection by targeting the amino acids 141-155 of the VP2 “puff” region, which belongs to the “southern rim” of the canyon (39). This VP2-specific antibody may interfere with the PSGL-1-mediated viral infection process by steric hindering. In addition, MAb JL2 inhibited EV-A71 infection with the inhibitory effect at the concentration of 0.1 g/ml, by preventingEV-A71 binding to SACRB2. Specifically, MAb JL2 not only binds to the residues 77–113,144–151, and 302-478 of human SCARB2, but also locks the configuration of SCARB2 (40). A crucial pH-dependent conformational shift of SCARB2’s helices 5 and 7 creates a lipid-transfer tunnel to facilitate the evacuation of a hydrophobic pocket factor from the virion, which is necessary for uncoating (41). At neutral pH, MAb Jl2 prevents EV-A71 uncoating by stabilizing the SCARB2 structure.

The murine antibody A9 is another representative attachment-blocking antibody, which demonstrated strong neutralizing activity against EV-A71 with an IC_50_ of 0.1 nM (42). The overlapping footprint of the A9-Fab included the VP1 C terminus (residues 289 to 294), VP3 “puff,” the BC loop, and VP3 α2 (residues 144 to 150), which are between the 2-fold and 3-fold axes. Since the overlapping regions of the epitopes cover the site where the virus binds SCARB2, MAb A9 prevents EV-A71 attachment by impeding the binding between EV-A71 and SCARB2, destroys the viral capsid structure, and destabilizes virions to cause genome release.

Additionally, certain anti-EV-A71 antibodies cause the uncoating and early release of the viral genome to neutralize the virus. Through the activation of genome release coupled with a conformational shift, converting infectious virions into A particles, MAb E18 mediated viral neutralization is initiated. Specifically, the binding sites for MAb E18 are found between the VP4-VP2-VP3-VP1 protomers, which encompass the binding of SCARB2 (43). As a result, MAb E18 neutralizes the virus by receptor-mimicking and sterically competing with the virus for receptor interactions.

Notably, in addition to helping virus attachment to host cells, certain receptors support viral uncoating. In particular, initial attachment during EV-A71 entrance is facilitated by HS, which is ubiquitously present on the surface of all mammalian cells, whereas SCARB2, which is largely found in the membranes of lysosomes and endosomes, enables viral uncoating. It is known that uncoating of EV-A71 takes place in endosomes and is related to the continuous acidification of the endosome. Neutralizing MAbs for the majority of non-enveloped viruses often focuses on one single step of entry. However, the MAb D5 can neutralize EV-A71 at both the pre- and post- attachment stages, with the IC_50_ of D5 of 0.324 μg/ml and 0.539 μg/ml (44, 45). The EV-A71 VP1 215-219 KQEKD amino acid, recognized by MAb D5, is present in the surface-exposed GH loop of VP1 and adjacent to the canyon area and entirely conserved across all EV-A71 subgenotypes. This binding site was within the VP1-6 site, which is crucial for the binding of SCARB2. Furthermore, the MAb D5 epitope was sterically positioned between the VP2-2 and VP3-4 sites, which indicated that MAbs might obstruct SCARB2 binding to these sites through steric hindrance (46). Investigation into the mechanisms of neutralization revealed that the internalization, subsequent uncoating, and RNA release after EV-A71 entrance were all prevented by MAb D5 at the post-attachment stage.

The host antibodies’ targets may only make up a small amount of the neutralizing epitopes on the EV capsids, which may only cover a tiny percentage of the exposed capsid. Linear neutralizing epitopes contain BC loop (97-105aa) (47), VP1-43 (48), VP1-145 (33, 49), EF loop (163-177aa), GH loop (208-225) (45) of VP1, VP2-28 (48) and the residues 141–146 of the VP2 EF loop (39, 49). Importantly, a conformational epitope associated with the SCARB2-binding site on the southern rim of the canyon is made up of the VP1 GH Loop and VP2 EF Loop. Additionally, prior investigations of murine cross-neutralizing mAb identified a conserved conformational epitope in the VP3 knob region in EV-A71 (50).

The majority of human antibody-recognized neutralizing epitopes were discovered to be conserved among EV-A71 viruses (51, 52). A panel of powerful antibodies was able to neutralize multi-genotype EV-A71 at concentrations under 100 ng/ml at both the serological and clonal levels, according to one investigation on human blood. Potent and broadly cross-reactive antibodies detecting new neutralizing epitopes on the floor and rims of the capsid canyon were isolated. However, the neutralization breadth and efficacy of antibodies that identified the 3- and 2-fold plateau epitopes on the margin of pentamer were limited. Additionally, only antibodies targeting the canyon rim showed inhibitory activity at the post-attachment stage with 10- to 100-fold greater concentrations than the EC_50_ at the pre-attachment stage (53).

Previous investigations have documented the cross-neutralizing antibody responses of infants and children against various subgenotypes of EV-A71 and CVA16 (54, 55). However, few broadly cross-neutralizing anti-EV antibodies have been identified. Analysis of the serological and monoclonal levels of neutralizing antibody responses demonstrates that the neutralizing epitopes are potentially correlated to the antibody immunity, which may contribute to the development of potent broadly cross-neutralizing anti-EV antibodies. A summary of reported EV-A71 neutralizing antibodies is shown in **Table 1**.

**Table 1:** Summary of enterovirus neutralizing antibodies.

EV	Mab name	Class	Origin (genus)	Isolation technology	Epitope type	Epitope Sequence	Mechanism	Potency
EV-A71	MA28-7 (38)	IgG	Mouse	Hybridoma	Conformational	VP1-145 aa and VP1-98, VP1-242, VP1-244	Block PSGL-1 and HS- mediated attachment	UN
	BB1A5 (39)	IgG	Mouse	Hybridoma	Linear	VP2 TESH (aa141-155)	Block PSGL-1-mediated attachment	neutralization titer of 1:32 Full protection: 60 mg/g
	JL2 (40)	IgG	Mouse	Hybridoma	Conformational	AA 77–113,144–151, and 302-478 of human SCARB2	Block SCARB2 2 and prevent virus uncoating	IC_50_: 0.66 μM
	A9 (42)	IgG	Mouse ascites (Fab fragment)	Hybridoma	Conformational	BC loop, aa 144-150 of α2 (VP3 “puff”), aa 289 -294 of VP1 C terminus	Block SCARB2-mediated attachment; Destabilizes virions to induce genome	IC_50_: 0.1 nM
	E18 (43)	IgG	Mouse	Chimeric	Conformational	VP4–VP2–VP3–VP1 protomers	Block SCARB2-mediated attachment, induce viral genome release	IC_50_: 5.2 nM
	D5 (44, 45)	IgG	Mouse	Hybridoma	Conformational	VP1 GH loop (aa 208-225)	Block internalization and subsequent uncoating and RNA release	IC_50_: 1.35 μM
	10D3 (50)	IgM	Mouse	Hybridoma	Conformational	VP3 Knob region, especially P59, A62, E67	Neutralize all EV-A71 subgenogroups A, B, C	neutralization titer: 1:64 to 1:256 Full protection: 10 mg/g
	51 (56)	IgM	Mouse	Hybridoma	Linear	VP1 GH loop (aa 215–219)	Neutralizing EV-A71 genotypes A, B1-B5, and C1-C5	neutralization titer: 1:1024
	2G8 (57)	IgM	Mouse	Hybridoma	Linear	VP1 GH loop KQEKD (aa 215–219), especially K218 and L220	UN	neutralization titer: 1:32
	M20 (58)	IgM	Mouse	Hybridoma	Linear	VP1 GH loop (aa 219-223), Y222 and G223	Inhibition of both the pre- and post-attachment of EV-As, involving entry, uncoating and RNA release. Neutralizing EV-A71, CVA16, CVA10, and CVA6	IC_50_: ~1 nM Full protection: 1 mg/g
	F1-hFc (59)	human IgG1 Fc fusion with Single Domain Antibody	Human-llama	Phage display	Conformational	VP3 (aa 46–60 and 16-130)	UN	EC_50_: 406 nM
	E18-F1 (60)	Single domain antibody armed chemeric IgG	Human	Antibody engineering	Conformational	Combination of E18 and F1 epitope	UN	EC_50_: 1.6 nM
CVA10	2G8 (61)	IgG	Mouse	Hybridoma	Conformational	South rim of the canyon surrounding the quasi- threefold axis, including VP1 C termini, VP2 EF loop, and VP3 AB loop	Mainly pre-attachment inhibitory effect	IC_50_: 0.2 μg/ml full protection: 30 μg/g
	M3-8 (62)	IgG	Mouse	Hybridoma	linear	2C protein, SLATGIIARA located in the ATPase domain	UN	Titer: 1:80000
CVA16	NA11F12 (63)	IgG	Mouse	Hybridoma	Linear	VP1	Neutralizing A, B1, B2 and C subgenotypes of CVA16	neutralization titer: > 1:1024 full protection: 0.1 μg/g
	18A7 (64)	IgG	Mouse	Hybridoma	Conformational	Discontinuous epitope spanning the DE loop and the HI loop of two adjacent VP1 capsid proteins	UN	IC_50_: 0.04 μg/ml
	14B10 (64)	IgG	Mouse	Hybridoma	Conformational	Conformational epitope on mature virions surrounding viral 3-fold and 2-fold axes	UN	IC_50_: 1.01 μg/ml
	NA9D7 (64)	IgG	Mouse	Hybridoma	Conformational	Conformational epitope on mature virions surrounding viral 3-fold and 2-fold axes	UN	IC_50_: 1.96 μg/ml
E30	6C5 (65)	IgG	Mouse	Hybridoma	Conformational	VP1 BC loop (E82, K83, V84, D86, E87, D89, Y91), VP1 DE loop (T130), VP1 EF loop (K156, E159), and the VP1 HI loop (T229)	Blocking viral binding with attachment receptor CD55 and uncoating receptor fcrn	IC_50_: < 30 nM
	4B10 (65)	IgG	Mouse	Hybridoma	Conformational	residues 137, 138, 159, 161, and 163 of VP2 EF loop, residues 260 and 268 of VP1 C-terminal loop, and residue 234 of VP3 C-terminus	Blocking viral binding with attachment receptor CD55 and uncoating receptor fcrn	IC_50_: < 30 nM
EV-D68	A6-1 (66)	IgG	Rhesus monkeys	Single b cell isolation	Linear	VP1 DE loop	Blocking the canyon region from binding with α2,6-linked sialic acids on the cellular surface through steric hindrance	IC_50_: 0.6 μg/ml for the KM strain and 1.57 μg/ml for the Fermon strain
	15C5 (67)	IgG	Mouse	Hybridoma	Conformational	VP2 BC loop and the VP3 BC loop from two adjacent protomers targeted by the heavy chain of Fab 15C5, and the AB, BC and HI loops of the same VP3 that were targeted by the light chain	Mimicking engagement by the functional receptor ICAM-5, blocks binding to host cells	IC_50_: 1.5 μg/ml
	11G1 (67)	IgG	Mouse	Hybridoma	Conformational	BC, DE, EF and HI loops and two β -strands of EV-D68 VP1	Binding to A-particle, blocks binding to host cells	IC_50_: 39.7 μg/ml
	2H12 (68)	IgG	Mouse	Hybridoma	Conformational	VP1 GH loop, VP2 EF loop, and VP3 C terminus	Blocking virus-receptor interactions, impairing virion integrity and inducing premature virion uncoating	IC_50_: 0.412 μg/ml
	8F12 (68)	IgG	Mouse	Hybridoma	Conformational	VP1 BC loop, VP1 GH loop, VP2 EF loop, and VP3 C-terminus	Blocking virus-receptor interactions	IC_50_: 0.004 μg/ml
	EV68-228 (69)	IgG	Human	Ebv-transformation and hybridoma	Conformational	VP1 DE loop and VP2 EF loop	UN	IC_50_: 0.32 ng/ml Full protection: 1μg/g
RVB14	C5 (70)	IgG	Mouse	Hybridoma	Linear	VP3	Inducing virus uncoating	UN
	YDF (71)	ScFv	Human	Antibody engineering	/	3C protease	Noncompetitively inhibits 3C peptidase activity	UN
	GGVV (71)	ScFv	Human	Antibody engineering	/	3C protease	Abolishes interaction between HRVB14 3C protease and its 5’ noncoding region of HRV genomic RNA	UN

UN, Unknown.

In recent years, it has been increasingly reported that CVA10 co-circulates with EV-A71 and CVA16, with CVA6 causing HFMD outbreaks in Asia, Africa, Europe, and North America. Although the amino-acid sequence identity of CVA10 with EV-A71 and CVA16 reaches ~69%, the receptor for viral entry into host cells is different, which makes CVA10 a KREMEN1-dependent subgroup of HEV-As (72). By immunizing mice with mature CVA10 virions, a hybridoma monoclonal antibody 2G8 was obtained. 2G8 exhibits high binding efficiency with all three forms of CVA10 virions, with potent neutralization activity in the invitro experiment (IC_50_ = 0.2 μg/ml) (61). 2G8 provided full protection against CVA10 infection both as a preventive and therapeutic strategy at a dose of 30 μg/g. In addition, ten times higher inhibitory efficacy in the pre-attachment assay (IC_50_ = 2.1 μg/ml) than post-attachment (IC_50_ = 22.7 μg/ml) was observed. The epitope of 2G8 maps to the south rim of the canyon surrounding the quasi-threefold axis, including VP1 C termini, VP2 EF loop, and VP3 AB loop, and is identical in all three types of particles, which explains the remarkable cross-reactivities of 2G8 against all three capsid forms.

Although CVA16 is one of the major pathogens causing HFMD, only several monoclonal antibodies against CVA16 that showed good potency and protective effect were reported, of which NA11F12 was reported as the most potent anti-CVA16 antibody. CVA16 monoclonal antibody NA11F12 obtained through immunization with the purified 190/CVA16 recognized a linear epitope on VP1 protein of CVA16A, CVA16B1, CVA16B2, and CVA16C, but not VP1 of EV-A71, while the detailed epitope of NA11F12 was not reported. The neutralizing titers of NA11F12 against different CVA16 subgenotype strains reached higher than 1:1024. Treatment of NA11F12 at 0.1 μg/g dose provided full protection in mice and the median effective dose (ED_50_) in newborn BALB/c mice was estimated as low as 0.0042 μg/g (63). 18A7, CVA16-specific neutralizing monoclonal antibody identified by He et al. neutralizes restricted strains of CVA16. The atomic structure showed that 18A7 binds to the viral 5-fold vertex of the CVA16 particles, including A-particles, empty particles, and mature virions of all three types, the epitope constitutes the DE loop and the HI loop of two adjacent VP1 (64). 14B10 and NA9D7, another two antibodies identified by the same group both recognize broad protective CVA16 epitopes, which bind mature virions only surrounding viral 2-fold and 3-fold axes, providing full protection against lethal CVA16 infection at a dose of 3 μg/g in neo (64). Besides, 8C4 has been discovered to protect neonatal mice from CVA16-induced death at 1-day post-infection at a dose of 10 μg/mouse (73). By using a phage display technique, Zhang et al. isolated a chimeric antibody that targets a highly conserved peptide in VP4 and cross-neutralized EV-A71 and CVA16 *in vitro*. But due to a lack of affinity maturation as often encountered by phage display, the neutralization capacity was relatively low, with the IC_50_ value higher than 60 μg/ml (74). Moreover, Shi et al. reported six conserved neutralizing linear epitopes within the VP1 of CVA16 (75). These epitopes include PEP32 (residues 94–108), PEP55 (residues 163–177), PEP71 (residues 211–225), PEP63 (residues 187–201), PEP91 (residues 271–285), and PEP37 (residues 109–123). Among these epitopes, PEP71 locates at a region overlapping with two linear epitopes of EV-A71, SP70 (residues 208–222) and VP1-43 (residues 211–220). The immunized sera targeting these epitopes showed cross-neutralizing activity against homologous and heterologous CVA16 strains, indicating the potential use of these peptides for the development of broadly protective CVA16 vaccine.

## 3 Anti- *Enterovirus B* antibodies

Currently, no approved vaccines are available for preventing EVBs infections. Echovirus 30 (E30), one serotype of the species EVB, has emerged as one of the most common pathogens of aseptic meningitis worldwide (76, 77). Two serotype-specific neutralizing antibodies against E30 were reported. 6C5 and 4B10 bind to E30 with high affinities at Kd values of 1.51 and 2.88 nM, respectively. Both intact IgG and Fab fragments of 6C5 and 4B10 could neutralize E30 with a 50% neutralizing concentration below 30 nM (65). No cross-reaction or neutralization reactions of 6C5 and 4B10 with other EVBs were detected. The epitope recognized by 6C5 mainly involves in VP1 BC loop, DE loop, EF loop, and the HI loop, which is located at the north rim of the canyon. The epitope of 4B10 mainly includes VP2 EF loop, VP1 C-terminal loop, and VP3 C-terminus that was inside the canyon. 6C5 and 4B10 exerted neutralizing activity by blocking viral binding to attachment receptor CD55 and uncoating receptor FcRn, and the two antibodies functioned both in the pre-attachment and post-attachment approach in a complementary manner. Additionally, 3A6 (78), a rat hybridoma monoclonal EV antibody that showed no neutralization activity to CVB1, recognized N-terminus of VP1 of several EVBs and also the EV-C representative Poliovirus 3, which was a good supplement for immunocytochemistry and immunohistochemistry study in the mouse model.

## 4 Anti- *Enterovirus D* antibodies

Enterovirus D68 (EV-D68) belongs to human enterovirus species D and is an emerging pathogen that causes respiratory illness and/or acute flaccid myelitis (AFM) (79, 80). The first reported potent EV-D68 antibody A6-1 was isolated from an EV-D68-infected rhesus macaque through single B cell sorting (66). The antibody protected suckling mice from EV-D68 intranasal infection at a dose of 30 mg per mouse. A6-1 achieved 100% inhibition of two EV-D68 strains, with IC_50_ values of 0.6 μg/ml for the KM strain and 1.57 μg/ml for the Fermon strain. F4-3, E2-2, and D7-4 mAbs isolated by this research group also showed neutralizing potency with IC_50_ in the range of 2–6 μg/ml. A6-1 binds to the DE loop of EV-D68 VP1, causing a steric hindrance that blocks the canyon region from binding with α2,6-linked sialic acids on the cellular surface (an EV-D68 receptor), and inhibits infections mainly through the pre-attachment inhibition effect.

Another two EV-D68-specific neutralizing antibodies, 15C5 and 11G1, obtained through EV-D68 immunized mice, exhibited different binding features and neutralizing mechanisms (67). 15C5 binds both mature virions and procapsids with high efficiency with an IC_50_ value of 1.5 μg/ml, while 11G1 showed weak binding affinity with an IC_50_ value of 39.7 μg/ml. A 100% survival rate was obtained in 1-day-old mice treated with 15C5 (60 μg/g). The epitope of 15C5 consists of the VP2 BC loop and the VP3 BC loop from two adjacent protomers, while the VP2 BC loop is targeted by the heavy chain of Fab 15C5, and the AB loop, BC loop, and HI loops of the same VP3 are targeted by the light chain. VP3 BC loop was critical for virus–antibody interactions, and mutation of L74S leads to resistance to 15C5. 11G1 interacts with BC loop, DE loop, EF loop, and HI loops, and two β -strands of EV-D68 VP1, mutations at position 229 (Q229K) or position 234 (T234A) of VP1 lead to 11G1-resistant. Structure analysis exhibited that 15C5 and 11G1 engage the capsid epitope at icosahedral three-fold and five-fold axes, respectively. In all, to block binding to host cells, 15C5 binds to all three forms of capsids and triggers the conformational transformation of mature virions to A-particles like that induced by binding with the functional receptor ICAM-5, whereas 11G1 recognizes the A-particle and exerts neutralization through a unique post-attachment mechanism.

2H12 and 8F12, obtained through immune mice with recombinant VLP of EV-D68 strain (US/MO/14-18950), showed a high binding affinity with VLP at nanomolar level, with IC_50_ values of 0.412 and 0.004 µg/ml against EV-D68 strain 18947, respectively (68). When H12 and 8F12 were mixed into a cocktail at the ratio of 1:1, the two antibodies complemented each other in neutralizing different EV-D68 strains, and the neutralization concentrations against 18947, 18953, and Fermon were 0.12, 0.24, and 1.95 µg/ml. A single dose (10 µg/g) of 2H12 and 8F12 for prophylactic efficacy reached 92% and 100% protection of the mice from lethal challenges. Using the cocktail for the treatment of strain 18947 or 18953 at 3 days post-infection (dpi), the survival rates reached 92% and 100%, respectively. The two antibodies bind to the south rim of the canyon around the two-fold axes, block the binding of EV-D68 to cellular sialic acid receptor via steric hindrance, and thus inhibit EV-D68 attachment. Additionally, 2H12 could also impair virion integrity and induce premature virion uncoating. Given the good neutralization potency and successful retainment of functions after human-mouse chimeric transformation, the 2H12/8F12 cocktail may serve as a good pan-EV-D68 therapy agent.

Using human B cell hybridoma technology, Vogt et al. reported a panel of human monoclonal antibodies targeting EV-D68, of which EV68-228, potently neutralizing cross strains of EV-D68with IC_50_ value of 0.32 ng/ml (69) and clade-specific mAb EV68–159 were selected. Cryo-electron microscopy revealed that EV68-228 bound around the five-fold axes, and recognized the classical immunogenic sites (NIms) VP1 DE loop andVP2 EF loop, while EV68–159 attached three-fold axes on virion particles, and interacted with E271, R272, and D185 on the C -terminal of VP1 and the VP3 N-terminal loop before the B-β strand. Notably, the two antibodies were lack of western blot activity due to their conformation-dependent epitope. EV68–228 exhibited good prophylaxis and treatment effect, completely inhibited virus titers in the blood and lungs, reduced pro-inflammatory cytokine levels, and protected AG129 mice from AFM disease.

## 5 Anti-Rhinovirus antibodies

Rhinoviruses (RVs) are the major cause of the common cold in humans. There are three species of RVs, RV-A, -B, and –C, which use different receptors including intercellular adhesion molecule 1 (ICAM-1), low-density lipoprotein receptor (LDLR), and the cadherin-related family member 3 (CDHR3) (11, 81). Dong et al. reported an RVB14-specific neutralizing antibody C5 that binds to RVB14 VP3 (70). The binding of C5 leads to particle expansion of the capsid to allow the release of the viral genome. Temperature dependent and molar ratio dependent (between 1:60 and 1:180 of virus-C5 Fab) manner were found in C5-induced virus uncoating. The epitope targeted by C5 Fab molecules was adjacent to the 3-fold proximal region on the outer surface of the virus, which is formed mainly by residues in the VP1 C terminus, VP2 C terminus, VP3 N terminus, VP3 BC loop, and VP3 HI loop.

## 6 Novel anti-EV antibodies

### 6.1 IgM antibodies

Besides the traditional IgG antibodies, the IgM antibody which harbors pentameric structure confers IgM high valency and polyreactivity can also be a potent neutralizing antibody. The potent IgM antibody 10D3 recognizes a conserved Knob region of VP3, which can neutralize all EV-A71 subgenogroups with a neutralization titer of 64 (genogroups A, B) to 256 (genogroup C) by using hybridoma cell supernatant (50). In addition, 10D3 conferred 100% passive protection in 2-week-old AG129 mice against EV-A71 lethal infection prophylactically at a dosage of 10 ug/g of body weight. The mAb51 is another potent IgM antibody that possesses neutralizing ability against A, B1-B5, and C1-C5 genotypes of EV-A71 with a neutralization titer of about 1024 *in vitro* and 100% passive protection of 10 μg/g of body weight *in vivo*. The antisera produced by the SP70 peptide, which is completely protected against CPE *in vitro* and 80% protected against EV-A71 infection *in vivo*, is identical to the mAb 51 that can neutralize. Although the two antibodies targeted the identical epitope on the VP1 capsid protein, covering amino acids 215-219, the IgG antibody mAb 53 lacked neutralizing efficacy both *in vitro* and *in vivo *(56). As a result, it was hypothesized that immunoglobulin isotypes could be crucial to the neutralization activity.

In a suckling mouse model, the IgM antibody mAb 2G8 neutralized EV-A71 with a neutralization titer of 1:30 and provided complete protection against the lethally EV-A71 challenge. 2G8 neutralizes EV-A71 by targeting the SP70 of VP1, especially the K218 and L220 primarily at the attachment stage (57). All the potential 2G8 epitopes were accessible on the surface of the mature EV-A71 virion, and SP70 was positioned inside the GH loops of VP1. These crucial residues were responsible for human serum neutralization following a natural EV-A71 infection. Notably, all EV-A71 subgenotypes shared K218 and L220, which enabled cross-neutralization capacity of 2G8. However, the amino acids of this epitope differ from other EV-As, bringing a limited neutralization breadth.

In our previous investigation, the mouse hybridoma technique was used to create the IgM antibody M20. M20 has broad and potent anti-EV-A neutralizing action, with IC_50_ values against EV-A71, CVA16, CVA10, and CVA6 within the range of nanomolar doses (58). Besides, M20 exhibits cross-protectivity in one-day-old ICR mice when provided at a dosage of 10 μg/g of body weight against infections with EV-A71, CVA16, CVA10, and CVA6. M20 recognizes DLEYG (219-223), especially Y222 and G223 as critical residues, relatively conserved across the EV-A isolates, which increases the potency and breadth of neutralization capacity. On the other hand, to demonstrate the superiority of IgM antibody, two chimeric antibodies: 20-IgM (based on M20) and its IgG isotype 20-IgG were constructed for comparison. Although targeting the same epitope, 20-IgM showed better potent neutralizing efficacy and stronger affinity against EV-A71, CVA16, CVA10, and CVA6 than 20-IgG. Its neutralizing mechanism includes inhibition of both the pre- and post-attachment of EV-As, involving entry, uncoating and RNA release. As compared with IgG, with better recognition and binding activities to EV-As via IgM’s five variable regions, IgM was able to block the interaction of the receptor, vicinity of the viral epitope, and even the adjacent interaction. The good neutralization efficacy, broad cross-reactivity, and strong binding ability of IgM antibodies make it a potent monoclonal antibody against EV-As infection.

### 6.2 Engineered IgG-like or bi-specific antibodies

Using antibody engineering technology, bi-specific antibodies are constructed to target different types of enteroviruses with enhanced potency and broad-spectrum neutralization activity. Given that EV-A71 and CVA16 are the major pathogenic agents of HFMD and often co-circulate during outbreaks, Zhou et al. constructed bi-specific antibodies targeting EV-A71 and CVA16. Among the four constructed bi-specific antibodies, the antibody constructed by the scFV-IgG (single-chain antibody fragment (scFv) fused to the termini of heavy or light chain) strategy showed higher binding affinity and neutralizing activity than DVD-IgG (dual variable domains IgG) forms. Bs(scFv)4-IgG-1, comprised of the scFv of CVA16-specific NA9D7 on the heavy chain and the scFv of EV-A71-specific CT11F9 on the light chain, exhibited remarkable cross-reactivity and neutralization capacity against EV-A71 and CVA16 than its parental antibodies and provided effective protection against lethal EV-A71 and CVA16 challenge in neonatal mice (82). The IC_50_ values for Bs(scFv)4-IgG-1 against EV-A71/52-3 and CVA16/190 were 1.01 and 0.59 μg/ml, respectively, and that against the B3, B5, C2, and C5 subgenotypes of EV-A71 strains and two B1b subgenotypes of the CVA16 strains ranged from 0.21 to 2.53 μg/ml. Importantly, Bs(scfv)4-IgG-1 showed a significant treatment effect at a half dose of that of a mixture of CT11F9 mAb and NA9D7 mAb against single infection or co-infection of EV-A71 and CVA16.

Single domain antibodies (sdAB), due to their small molecular weight, strong stability, and easy recombination have been an attractive area for therapeutic antibody research recently. One llama-derived novel sdAB, F1, was reported to protect against EV-A71 infection both *in vitro* and *in vivo* (59). F1 recognizes a novel conformational epitope located at the highly conserved region of VP3. To compare valency effect, F1 was further engineered to bivalent sdAb F1-hFc and tetravalent F1×F1-hFc forms, while the tetravalent form exhibited at least 5.8-fold higher neutralization activity against EV-A71 than that of the bivalent sdAb F1-hFc and provided better protection in hSCARB2 transgenic mouse model with half doses as compared to F1-hFc. In another study done by this group, by fusing F1 to the light chain of an EV-A71-specific neutralizing antibody E18 at the C terminus via a G4SG3S linker, the obtained bi-specific IgG-like antibody E18-F1 exhibited enhanced binding and improved antiviral activity to EV-A71 compared with original antibodies (60). The binding signals of E18-F1 were more than two-fold higher than the original E18 antibody, and the neutralization EC_50_ of E18-F1 (1.6 nM) was lower than that of E18 IgG (5.2 nM) and F1-hFc (406 nM). In addition, pre-incubation with E18-F1 at 10 nM led to more than 99.5% reduction of viral loads. Moreover, E18-F1 (200 μg/mouse) protected hSCARB2 transgenic mice from paralysis challenged with a lethal dose of EV-A71. Therefore, bi-specific antibodies may serve as a promising agent for EV-A71 treatment.

### 6.3 Non-structure protein targeting antibodies

Besides the structure protein, the non-structure protein can also be a target for enterovirus neutralizing antibodies. M3-8, a monoclonal antibody targeting the 2C protein of CVA10 obtained by the hybridoma method, exhibited a high antibody titer of 1:80000 (62). M3-8 recognizes a linear epitope (SLATGIIARA) located in the ATPase domain in the 2C protein, especially G140, I141, I142, and R144, which is conserved in most EV-A species except CVA4, CVA14, EV-A92, and EVA-125. This made M3-8 potential for the diagnosis or the antiviral therapies development.

In addition, the 3C protease which is indispensable in the virus life cycle has also become the focus of enterovirus antiviral development. Two scFvs, YDF and GGVV, effectively inhibited HRVB14 proliferation by interfering with 3C protease activity (83). YDF noncompetitively inhibited 3C endopeptidase activity by an allosteric effect. GGVV blocked the interaction between HRVB14 3C protease and its 5’ noncoding region of HRV genomic RNA, thereby interfering with genome replication of HRV14. As 3C protease is crucial to the life cycles of rhinoviruses and other enteroviruses, as well as the conserved GGVV epitope presented in HRV14, HRV2, CVA16, CVB3, EV-A71, and enterovirus 93 (EV93), 3C protease of enterovirus, may serve as a broad-spectrum target for antiviral inhibitor development (84).

Recently, two EVA-71 3D^pol^ targeting monoclonal antibodies, 3A12 and 2A10, obtained by the hybridoma method, were reported to efficiently suppress virus replication through inhibition of the RNA elongation activity of 3D^pol^. Delivery of the two antibodies provided partial protection in neonatal mice. Therefore, the antibody against nonstructural protein of enteroviruses could also be a potential antiviral therapeutic drug.

## 7 Future strategies for NPEVs antibodies

Enteroviruses are a large family that lead to infections all over the world. Due to the high diversity of enterovirus serotypes, a broad-spectrum protective vaccine has not been developed yet. As an integral part of pathogen prevention and control, neutralizing antibodies, which play a key role in mediating *in vivo* protection, could serve as good complementary weapons to vaccines.

**Figure 1:**
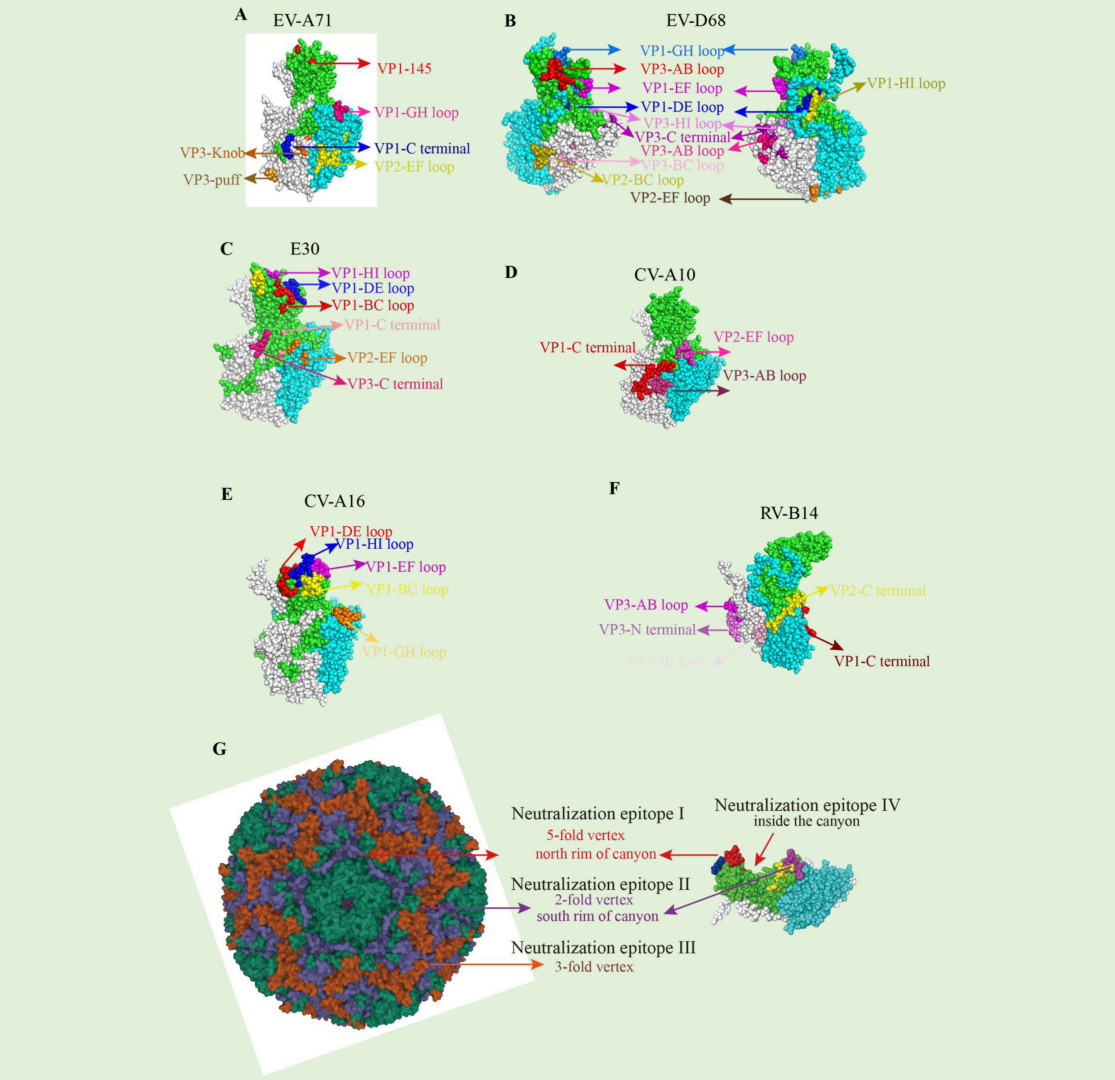
Neutralizing epitopes of Enterovirus. **(A)** Mapping of neutralizing antibodies against EV-A71. The naturalizing epitopes are located as VP1(EF, GH loop, and C-terminal), VP2-EF loop, and VP3 (knob and puff region), based on the EV-A71 (PDB 5ZUF). **(B)** Mapping of neutralizing antibodies against EVD68. The most naturalizing epitopes are located at VP1(DE, EF, GH, HI loop), VP2 (BC, EF loop), and VP3 (AB, BC, HI loop, and C-terminal), based on the EV-D68 (PDB 6CSH). **(C)** Mapping of neutralizing antibodies against E30. The most naturalizing epitopes are located at VP1(BC, DE, HI loop, and C-terminal), VP2 EF-loop, and VP3 C-terminal, based on the E30 (PDB 7C9T). **(D)** Mapping of neutralizing antibodies against CV-A10. The most naturalizing epitopes are located at VP1 C-terminal, VP2 EF-loop, and VP3 AB-loop, based on the CV-A10 (PDB 6AD1). **(E)** Mapping of neutralizing antibodies against CV-A16. The most naturalizing epitopes are located at VP1(BC, DE, EF, GH, HI loop), based on the CV-A16 (PDB 5C9A). **(F)** Mapping of neutralizing antibodies against RV-B14. The most naturalizing epitopes are located at VP1 C-terminal, VP2 C-terminal, VP3(N-terminal, and AB, HI loop) based on the RV-B14 (PDB 5W3O). **(G)** Classification of neutralization epitope of enterovirus.

Enterovirus-specific antibodies exhibited different features and activities. Understanding the affinity and activity, neutralizing mechanism, protective effect, and the epitope, especially the conservative region, will profit the pursuit of novel vaccine design, such as peptide vaccines and conservative anti prototype nanoparticle vaccines. A good example is that the chimeric virus-like particle (VLP) vaccine displaying conserved EV-A71 epitopes (SP55 and SP70) elicited carrier- and epitope-specific neutralizing antibodies and protective effect in mice (53). Several neutralizing epitopes for enteroviruses which are distributed across four major patches have been discovered by the cryo-EM study of virion-NAbs complexes, as discussed by Wang et al. (65), here we show the epitopes in **Figure 1**: (1) the north rim of the canyon around the 5-fold axis, which is recognized by anti-E30 6C5 (65), anti-CVA6 1D5 (85), and anti-EV-D68 11G1 (67); (2) the south rim of the canyon surrounding the 2-fold axis targeted by anti-EV-A71 D6 (42), anti-EV-A71 D5 (44), and anti-CVA10 2G8 (61); (3) epitopes near the 3-fold axis, for example, those targeted by anti-EV-A71 E18 (43), anti-EV-A71 A9 (42), anti-EV-D68 15C5 (67) and anti-HRVB14 C5 (70); (4) epitopes inside the canyon, defined by anti-E30 4B10 (65). The antibody-virus interaction at structural regions such as these sites series may be essential for design efficient enterovirus vaccines.

The antiviral potency exerted by isolated neutralizing antibodies for enteroviruses varied a lot on the whole, of which only a few of them showed good potency but with restricted cross-activity, which may be a limitation for possible treatment in severe cases. The low access of broad-spectrum neutralizing antibodies against EVs can be explained by the relatively few conservative structural features and the different entry mechanisms of varied viral subtypes. On the other hand, a series of antibodies’ neutralizing mechanisms have been identified including blocking receptor-mediated attachment to cells, stabilization or destabilization of the capsid, interfering with uncoating and genome releasing, and inducing of virion aggregation (42, 86, 87), which inhibit infection at different stages of virus replication, either at pre-attachment or post-attachment or both. Based on the deep-going study of enteroviruses antibodies, the combination use of antibodies targeting viral protein and antibodies interfering with receptors might provide better inhibitory activity. It is necessary to consider the synergistic effect using antibody engineering techniques to construct bi-specific or multi-specific antibodies that can target different epitopes of multiple enteroviruses with improved antiviral breadth compared to parental antibodies. Such cases have been reported in HIV-1 treatments with combined or multi-specific neutralizing antibodies (88, 89), and EV-A71-CA16 bi-specific antibodies (60, 82), which could elevating up to 5 to 10 fold of cross-neutralization efficacy. Also, design of the antibody structure for the bi-specific antibody is also important and still worth exploring, as antibody in Bs(scFv)4-IgG structure or DVD-IgG structure exhibited different potencies. Such antibodies need to be based on antibody spectrum, neutralizing mechanism, and screening method. Given the expansive diversity and continuously evolving enteroviruses, the broad-spectrum is of priority for future antibody development.

The neutralizing activity is closely related to antibody affinity and epitope. Bivalent binding or even tetravalent binding could contribute to significantly stronger neutralization potency of IgG over Fab, as observed in the study that anti-EV-A71 D5-IgG and F1×F1-hFc antibodies showed higher potency than D5-Fab and F1-hFc (46, 59). Moreover, the isotype of the antibody could also cause differences in neutralizing ability. IgM antibodies are pentameric or hexameric, their polyvalency allows for high avidity binding and efficient engagement of complement to induce complement-dependent cell lysis (CDC). Anti-EV-A71 20-IgM exhibited much higher neutralization activity and provide full protection from clinical symptoms at a lower dose than that of the IgG form (58). Our study on anti-Rabies antibodies also showed that IgM isotype antibodies were beneficial to enhance the binding and neutralizing potency of antibodies targeting linear epitopes (data not published). The observation that the high antiviral activity correlated with the high affinity of pentavalent IgM antibodies suggests that antibody isotype modification based on antibody affinity is a feasible strategy to improve epitope binding and neutralizing activities (57, 58). Recently, an adaptive multi epitope targeting enhanced affinity platform (AMETA) based on IgM backbone exhibited exponentially enhancement of antiviral potency by forming high affinity nanoantibody IgM complexes (90). Compared with monovalent nanoantibodies, the neutralizing activity of this complex was increased by about 500 times on average. Its multivalent IgM backbone has flexible structural dynamics, allowing it to bind efficiently in a variety of pathogen geometries. Application of this method on anti-SARS-CoV-2 antibody achieved ultrapotent, broad, and durable efficacy against sarbecoviruses, together with robust preclinical results. In a word, as antibody-mediated protection may serve as a powerful strategy against enteroviruses disease outbreaks, polyvalency with high potency should be the direction for antibody isolation and generation in the future.

At present, monoclonal neutralizing antibodies against enteroviruses are mainly obtained by murine hybridoma technology, which require a lot of screening and are time-consuming. Although antibody humanization technology can largely solve the immune rejection of mouse-derived antibodies, the fully human antibody may be safer and more effective. By using sing cell flow cytometry or microfluidic sorting and gene amplification, single B cell sequencing and synthesis technology, high-throughput paired antibody genes from human memory B cells or stimulated plasma cells could be obtained for investigation. However, the disadvantages of doing so are also obvious, involving human ethical issues, biosafety issues, and human blood samples especially blood from children suffered from enteroviruses with high neutralizing potency are very precious and difficult to obtain, while mouse derived samples can be obtained by immunizing animals at a lower cost. Moreover, by adopting antibody engineering technology, bi-specific or multi-valent antibody could be obtained with improved potency, but a more complex purification processes as well as potential safety issues need to be well addressed before their therapeutic application. On the other hand, considering the transmission route of enteroviruses in the respiratory tract, in addition to conventional intramuscular injection, mucosal administration of neutralizing antibodies is an acceptably safe and tolerable strategy for short and medium-term prevention for the future application possibly.

Currently, there are no approved antiviral therapies against enteroviruses infection. Recent advances made in the field of neutralizing antibodies will likely be important hints against disease control. Step forward, the development of new antibodies would be essential to provide more candidates against enteroviruses.
